# RACIAL DIFFERENCES IN NON-COGNITIVE SYMPTOMS IN ALZHEIMER’S DISEASE AND CAREGIVER DEPRESSION

**DOI:** 10.1093/geroni/igz038.3150

**Published:** 2019-11-08

**Authors:** Weizhou Tang

**Affiliations:** University of South Carolina, Columbia, South Carolina, United States

## Abstract

Behavioral and psychological symptoms (BPS) represent a heterogeneous group of non-cognitive symptoms occurring in persons with Alzheimer’s disease (PwAD), and they are often associated with negative outcomes for AD caregivers. Evidence indicates differences in caregivers’ mental health across race/ethnic groups. However, there is a lack of research that compares racial differences in BPS in PwAD. This study aims to compare racial differences in BPS in PwAD and caregiver depression. The study analyzed data collected from the South Carolina AD Registry in 2010. The survey used in the interview included measures of caregiver depression, caregiver burden, PwAD’s non-cognitive symptoms, caregiving competence, caregiver distress, and demographics. The final analysis focused on 635 African-American (n=313) and white (n=322) caregivers. Mann-Whitney U-tests, Chi-square tests, and multiple linear regression were conducted. Among all PwAD, higher percentage of whites than African Americans exhibited apathy/indifference (67.52% vs 52.44%, p=.0001), depression/dysphoria (61.54% vs 44.59%, p<.0001), and anxiety (45.08% vs 29.64%, p<.0001). In terms of both frequency and severity of BPS, whites had significantly higher BPS score (Mean=35.49, SD=24.75) than African Americans (Mean=28.13, SD=23.97; p<.0001). Mean comparisons indicated significant group differences in caregiver depressive symptoms between white caregivers (mean=11.89, SD=6.90) and African-American caregivers (mean=9.41, SD=5.77). However, there were no racial differences in the relationship between BPS in PwAD and caregiver depression. The findings of this study highlight the importance of developing more effective and targeted treatment options and therapies for neuropsychiatric symptoms and delivering cultural relevant education programs/interventions to ethnic groups.

